# Ectopic Molar Pregnancy: Diagnostic Efficacy of Magnetic Resonance Imaging and Review of the Literature

**DOI:** 10.1155/2016/7618631

**Published:** 2016-08-25

**Authors:** Yasushi Yamada, Satoshi Ohira, Teruyuki Yamazaki, Tanri Shiozawa

**Affiliations:** ^1^Department of Obstetrics and Gynecology, Shinshu University School of Medicine, 3-1-1 Asahi, Matsumoto 390-8621, Japan; ^2^Department of Obstetrics and Gynecology, Iida Municipal Hospital, 438 Yawatamachi, Iida 395-8502, Japan

## Abstract

Ectopic molar pregnancy is extremely rare, and preoperative diagnosis is difficult. Our literature search found only one report of molar pregnancy diagnosed preoperatively. Moreover, there is no English literature depicting magnetic resonance image (MRI) findings of ectopic molar pregnancy. We report a case of ectopic molar pregnancy preoperatively diagnosed using MRI. A literature review of 31 cases of ectopic molar pregnancy demonstrated that lesions have been found in the fallopian tube (19 cases, 61%), ovary (5 cases, 16%), cornu (3 cases, 10%), peritoneum (2 cases, 6%), uterine cervix (1 case, 3%), and cesarean scar (1 case, 3%). Abdominal pain and abnormal vaginal bleeding were reported in 70% and 61% of the patients, respectively. Twenty-one cases (67%) presented with rupture and hemoperitoneum. All patients underwent surgical resection or dilatation and curettage. Methotrexate therapy was performed in one case because residual trophoblastic tissue was suspected. A second operation was performed in one case of ovarian molar pregnancy because serum hCG levels increased again after primary focal ovarian resection. No patients developed metastatic disease or relapsed. These findings suggest the prognosis of ectopic molar pregnancy to be favorable.

## 1. Introduction

Gestational trophoblastic disease (GTD) consists of hydatidiform mole, choriocarcinoma, placental site trophoblastic tumor, and epithelioid trophoblastic tumor. Because the majority of GTD cases occur in the uterus, ectopic molar pregnancy is extremely rare. Gillespie et al. estimated that the incidence of ectopic GTD is 1.5 per one million births in the UK [1]. Preoperative diagnosis of ectopic molar pregnancy is difficult, and our literature search found only one report of molar pregnancy diagnosed preoperatively [2]. Moreover, there is no English literature depicting magnetic resonance image (MRI) findings of ectopic molar pregnancy. Here, we report the first case of ectopic molar pregnancy preoperatively diagnosed using MRI, with a review of the literature.

## 2. Literature

We performed a review of all ectopic molar pregnancy cases published in English and Japanese between 1960 and 2014. All studies were obtained from Medline using the terms “ectopic molar pregnancy”, and from references of the articles. All articles without an abstract or with unavailable full text were excluded. We identified 26 articles reporting 31 cases of ectopic molar pregnancy [2–27] (Table 1).

## 3. Clinical Case

**Figure 1:**
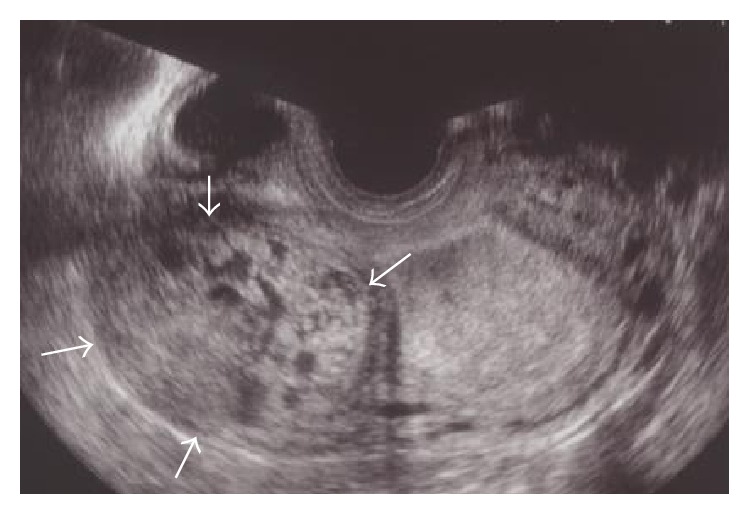
Transvaginal ultrasonographic image at 8 weeks of gestation. A hyperechoic mass (5 cm) in the right cornu containing multiple vesicles (arrows).

**Figure 2:**
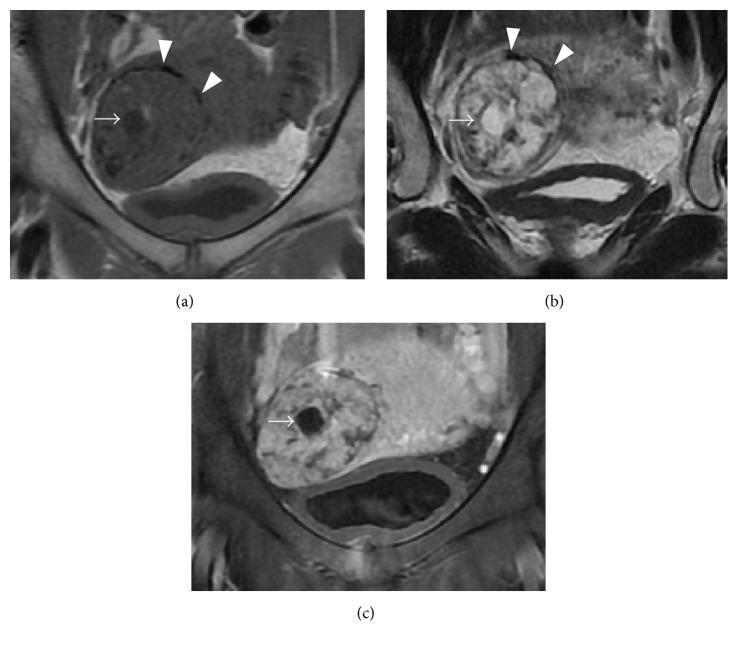
Coronal MRI images. (a) T1-WI shows an isosignal intensity mass in the right cornu. The mass includes a cyst (arrow) and shows low signal intensity. Several flow voids (arrow heads) are observed at the edge of the mass. (b) T2-WI. The mass and cyst (arrow) show high signal intensity. Several flow voids (arrow heads) are observed at the edge of the mass. (c) Gadolinium-enhanced and fat-suppressed T1-WI demonstrates a well-enhanced mass and clear margins. Arrow indicates the cyst.

We recently observed a 33-year-old, gravida 3 para 2, woman who visited our hospital with a complaint of amenorrhea for 8 weeks and 3 days since her last menstrual period. Her blood pressure was 104/76 mmHg, with pulse of 68 beats per minute. Her abdomen was soft and she had no tenderness on palpation. On vaginal examination, the uterus was asymmetrically enlarged. Transvaginal ultrasonography (TVUS) revealed an empty endometrial cavity and right cornual hyperechoic mass (5 cm) with multiple vesicles (Figure 1). Serum beta human chorionic gonadotropin (*β*-hCG) level was 66,400 ng/mL. Because molar ectopic pregnancy was suspected and her vital signs were stable, MRI was performed. MRI revealed a 5 cm mass on the right cornu, of isosignal intensity on T1-weighted images (T1-WI) and high signal intensity on T2-weighted images (T2-WI). The mass included vesicles with low signal intensities on T1-WI and high signal intensities on T2-WI, suggesting hydropic villi. The mass showed strong gadolinium contrast enhancement, and its margins were clear. Several flow voids were observed at the edge of the mass (Figure 2).

**Figure 3:**
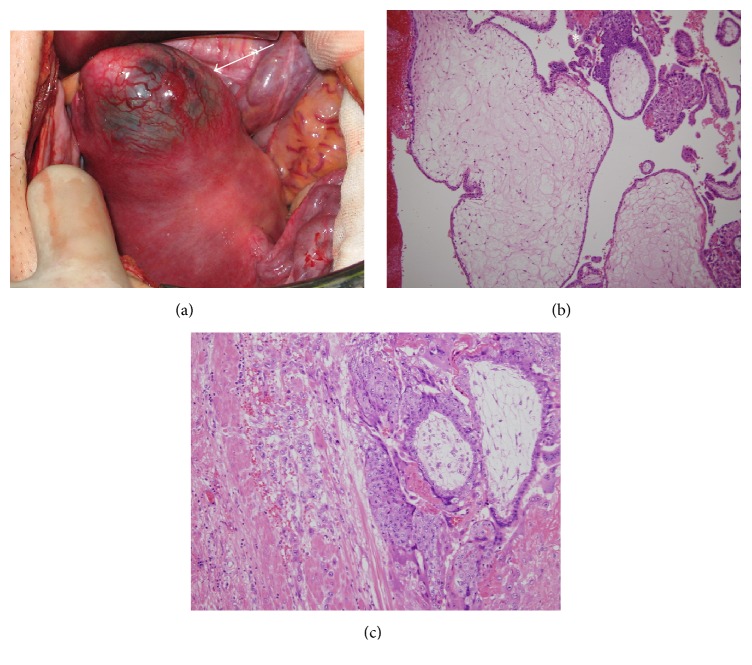
(a) Photograph of the uterus during laparotomy. A dark-blue mass is seen in the right cornu, with increased vascularity (arrow). (b) Enlarged hydropic villi and focal trophoblastic proliferation (asterisk) are observed. There is no cistern formation. A few proliferating stromal cells are observed but degeneration is not noted. (c) Invasion of villi and trophoblasts to the uterine myometrium. The trophoblastic proliferation is variable.

According to these findings, a preoperative diagnosis of ectopic molar pregnancy in the right uterine cornu was made. Because the patient no longer had any wish for a baby, an abdominal hysterectomy was performed. We chose not a laparoscopic surgery but a laparotomy to avoid rupture of enlarged uterine cornu during removing of the uterus through the vagina. On laparotomy, a dark-blue mass with increased vascularity in the right uterine cornu was noted (Figure 3(a)). Both adnexa were normal, and there was no hemoperitoneum. Total abdominal hysterectomy was performed because the patient and her husband did not wish to preserve fertility. Grossly, cut sections of the uterus showed a dark-red 4 cm mass with small vesicles in the right cornu. The uterus had no malformation such as unicornuate or bicornuate uterus. On pathology, chorionic villi with focal trophoblastic proliferation and hydropic change were observed. There was no cistern formation. A few proliferating stromal cells were observed but degeneration was not noted (Figure 3(b)). Invasion of trophoblasts to the myometrium was noted (Figure 3(c)). The postoperative diagnosis was ectopic invasive mole in the right cornu. Systemic computed tomography was performed after operation and revealed no metastatic lesion. The patient was followed up weekly or biweekly, and her *β*-hCG level was negative 8 weeks postoperatively. The patient has been free from relapse for 60 months.

## 4. Results (Table 1)

Of the 31 cases reviewed, the mean age was 31.3 years (20 to 44 years), and the lesions were found in the fallopian tube (19 cases, 61%), ovary (5 cases, 16%), cornu (3 cases, 10%), peritoneum (2 cases, 6%), uterine cervix (1 case, 3%), and cesarean scar (1 case, 3%). Abdominal pain and abnormal vaginal bleeding were reported in 70% and 61% of the patients, respectively. Twenty-one cases (67%) presented with rupture and hemoperitoneum. Serum *β*-hCG levels in 12 cases and serum hCG levels in 8 patients ranged within 3.5–404,000 mIU/mL and 3,454–165,000 mIU/mL, respectively. All patients underwent operation or dilatation and curettage. A second operation was needed in one ovarian molar pregnancy case because serum hCG levels increased again after primary focal ovarian resection. Methotrexate therapy was performed in one case because residual trophoblastic tissue was suspected. None of the patients developed metastatic disease or relapsed.

## 5. Discussion

Preoperative diagnosis of ectopic molar pregnancy is difficult, and we found only one reported case. Asseryanis et al. preoperatively detected a left tubal molar pregnancy using transvaginal color-flow Doppler, revealing an arteriovenous shunt flow of both the tumor and myometrium [2]. However, the efficacy of transvaginal color-flow Doppler in the diagnosis of ectopic molar pregnancy remains controversial [28]. We suspected cornual molar pregnancy because transvaginal ultrasonography revealed a mass with small vesicles in the right cornu, which is a typical finding of molar pregnancy. MRI revealed a right cornual mass with isosignal intensity on T1-weighted images (T1-WI) and high signal intensity on T2-weighted images (T2-WI). In addition, small vesicles in the mass showed low signal intensities on T1-WI and high signal intensities on T2-WI, which suggested hydropic villi. Distinguishing between ectopic molar pregnancy and choriocarcinoma or “ordinary” ectopic pregnancy is important. Ha et al. reported four important MRI findings for the differential diagnosis between uterine choriocarcinoma and uterine invasive mole: (i) the tumor margin is well-defined in choriocarcinoma and ill-defined in invasive mole; (ii) the hyperintensity pattern on T1-WI is nodular in choriocarcinoma and scattered in invasive mole; (iii) intratumoral vascularity is absent or minimal in choriocarcinoma due to severe central necrosis and hemorrhage, whereas intratumoral vascularity is increased and the tumor is densely enhanced in invasive mole; and (iv) invasive mole has molar tissue-like tiny cystic lesions within the mass [29]. Although the disease site differed, their suggestions may be useful for diagnosing ectopic molar pregnancy. In our case, molar tissue-like tiny cystic lesions, intratumoral hypervascularity, and dense enhancement were observed. We believe that MRI is a powerful tool for diagnosis of ectopic molar pregnancy. However, there may have been several cases in which MRI was not performed due to shock induced by rupture.

The rate of rupture and hemoperitoneum in cases of molar ectopic pregnancy rupture was 67%. Berlingieri et al. and Frates et al. reported rates of ruptured normal ectopic pregnancy of 29.5% and 25.2%, respectively [30, 31], demonstrating that the rate of molar ectopic pregnancy rupture was significantly higher than that of normal ectopic pregnancy. This may be due to the higher invasive ability of trophoblasts in gestational trophoblastic disease compared with trophoblasts in normal pregnancy.

The serum *β*-hCG levels in 12 cases and serum hCG levels of 8 patients ranged within 3.5–404,000 mIU/mL and 3,454–165,000 mIU/mL, respectively. Frates et al. reported that the serum hCG levels of 225 normal ectopic pregnancy ranged within 7–107,949 mIU/mL [31]. Tasha et al. reported 18 cases of ectopic gestational trophoblastic disease in 100 cases of ectopic pregnancy. The hCG levels of normal ectopic pregnancy were 1,256–13,494 mIU/mL, partial mole 6,642–15,678 mIU/mL, and complete mole 7,920–24,733 mIU/mL. Furthermore, cases of intrauterine molar pregnancy are known to have higher hCG levels than normal pregnancies. Although Chauhan et al. suggested that implantation in the fallopian tube might preclude adequate vascularization and lead to low hCG levels in ectopic molar pregnancy [6], these reports suggested that ectopic molar pregnancy cannot be distinguished from normal ectopic pregnancy by hCG levels alone. Because none of the patients developed metastatic disease or relapsed, the prognosis of molar ectopic pregnancy is suggested to be favorable.

## Figures and Tables

**Table 1:** Thirty-one cases and the current case on ectopic molar pregnancy.

Case number	Author	Age (years)	Gestation (weeks)	Site	Symptom	hCG type	hCG level (mIU/mL)	Rupture	Preoperative diagnosis	Treatment
1	Asseryanis et al. [2] (1993)	27	16	Left tube	Pelvic mass	*β* hCG	3.5	−	Ectopic molar pregnancy	Left tubal resection
2	D'Aguillo et al. [3] (1982)	24	15	Right ovary	Abdominal pain	*β* hCG	44000	−	Right tubal pregnancy	RSO
3	Chase et al. [4] (1987)	38	8	Right tube	Amenorrhea	*β* hCG	83	−	Ectopic pregnancy	Laparoscopic right tubal resection
4	Chapman [5] (2001)	35	9	Cervix	Vaginal bleeding	*β* hCG	90181	−	Cervical pregnancy	D&C + laparoscopy
5	Chauhan et al. [6] (2004)	27	6	Right tube	Abdominal pain	*β* hCG	406	−	Right tubal pregnancy	Laparoscopic right tubal resection
6	Wu et al. [7] (2006)	31	7	Cesarean scar	Abdominal pain Vaginal bleeding	*β* hCG	61798	−	Missed abortion	D&C × 2
7	Chauhan et al. [8] (2006)	40	12	Left cornua	Abdominal pain Vaginal bleeding	*β* hCG	2905	−	Unknown	TAH
8	Tulon et al. [9] (2010)	30	7	Left tube	Abdominal pain Vaginal bleeding	*β* hCG	5308	+	Ruptured ectopic pregnancy	Left tubal resection
9	Hwang et al. [10] (2010)	41	12	Left cornua	Vaginal bleeding	*β* hCG	57738	−	Ectopic pregnancy	Laparoscopic left cornual resection
10	Juan [11] (2013)	20	8	Left tube	Abdominal pain	*β* hCG	6984	+	Left tubal pregnancy	Laparoscopic left tubal resection
11	Mbarki et al. [12] (2015)	32	6	Left tube	Abdominal pain Vaginal bleeding	*β* hCG	404000	+	Left tubal pregnancy	Laparoscopic left tubal resection
12		37	7	Left tube	Abdominal pain Shock vital	*β* hCG	290600	+	Ruptured ectopic pregnancy	Laparoscopic left tubal resection
13	Jock et al. [13] (1981)	27	12	Left ovary	Amenorrhea	Serum hCG	165000	+	Ovarian choriocarcinoma	LSO + OM + D&C
14	Zite et al. [14] (2002)	Unknown	12	Right cornua	Abdominal pain	Serum hCG	97000	+	Intrauterine mole + ovarian bleeding	Right cornual resection + D&C
15	Mohamed and Sharma [15] (2003)	32	Unknown	Right tube	Abdominal pain Vaginal bleeding	Serum hCG	7823	+	Ectopic pregnancy	Right tubal resection
16	Church et al. [16] (2008)	29	6	Left ovary	Abdominal pain Vaginal bleeding	Serum hCG	3584	+	Left tubal pregnancy	LSO
17	Leung et al. [17] (2010)	38	Unknown	Uterus + right ovary	Vaginal bleeding	Serum hCG	54000	+	Intrauterine mole	D*＆*C → right ovarian resection
18	Bousfiha et al. [18] (2012)	32	6	Left tube	Abdominal pain Vaginal bleeding	Serum hCG	3454	−	Ectopic pregnancy	Laparoscopic left tubal resection
19	Sehn et al. [19] (2013)	20	Unknown	Left ovary	Abdominal pain Vaginal bleeding	Serum hCG	100355	+	Unknown	Laparoscopic left ovarian resection → laparoscopic LSO
20	Ota et al. [20] (2014)	23	8	Peritoneum	Abdominal pain Shock vital	Serum hCG	8000	+	Ruptured ectopic pregnancy	Laparotomy
21	Ikuma et al. [21] (1992)	44	11	Left tube	Vaginal bleeding	Urine hCG	1600	+	GTD	MTX 1corse → ATH + LSO → MTX 1corse
22	P. Dumitrescu and A. Dumitrescu [22] (1960)	28	Unknown	Peritoneum	Unknown	Not performed	—	+	Ectopic pregnancy	Laparotomy
23	Westerhout Jr. [23] (1964)	32	8–10	Left tube	Abdominal pain Vaginal bleeding Shock vital	Not performed	—	+	Unknown	LSO
24	Pour-Reza [24] (1974)	36	Unknown	Left tube	Abdominal pain Vaginal bleeding	Not performed	—	+	Ectopic pregnancy	LSO
25	Farrukh et al. [25] (2007)	27	Unknown	Right tube	Abdominal pain Vaginal bleeding	Not performed	—	+	Ectopic pregnancy	Laparoscopic right tubal resection
26	Samaila et al. [26] (2009)	20	Unknown	Tube	Abdominal pain Vaginal bleeding	Not performed	—	+	Unknown	Laparoscopic tubal resection
27		28	Unknown	Tube	Abdominal pain Vaginal bleeding	Not performed	—	+	Unknown	Laparoscopic tubal resection
28		33	Unknown	Tube	Abdominal pain Vaginal bleeding	Not performed	—	+	Unknown	Laparoscopic tubal resection
29		35	Unknown	Tube	Abdominal pain Vaginal bleeding	Not performed	—	+	Unknown	Laparoscopic tubal resection
30		37	Unknown	Tube	Amenorrhea	Not performed	—	+	Unknown	Laparoscopic tubal resection + LM
31	Yakasai et al. [27] (2012)	35	12	Left tube	Abdominal pain	Not performed	—	+	Left tubal pregnancy	Left tubal resection
32	Current case	33	8	Right cornua	Amenorrhea	*β* hCG	66400 (ng/mL)	−	Ectopic molar pregnancy	TAH

hCG: human chorionic gonadotropin; TAH: total abdominal hysterectomy; LSO: left salpingo-oophorectomy; RSO: right salpingo-oophorectomy; D&C: dilatation and curettage; OM: omentectomy; LM: laparoscopic myomectomy; MTX: methotrexate; GTD: gestational trophoblastic disease.
